# An unusual course of the vertebral artery posterior to the nerve root in the inter-transverse space: a cadaveric study

**DOI:** 10.1186/s13037-015-0072-7

**Published:** 2015-05-14

**Authors:** Ali Nourbakhsh, Jinping Yang, Bruce Ziran, Kim J. Garges

**Affiliations:** Atlanta Medical Center, 303 Parkway Dr. NE, 30312 Atlanta, GA USA; Department of Orthopaedic Surgery & Rehabilitation, University of Texas Medical Branch, 301 University Boulevard, Galveston, TX USA; Gwinnett Medical Center, 575 Professional Dr., Suite 360, 30046 Lawrenceville, GA USA; Houston Physician’s Hospital, 333 North Texas Avenue, 77598 Webster, TX USA

**Keywords:** Cervical, Vertebral artery, Nerve root

## Abstract

**Background:**

The V2 segment of the vertebral artery is very vulnerable to injury during cervical spine surgery. The incidence of vertebral artery injury during anterior cervical spine procedures is reported to be 0.22–2.77 %. This is partially due to its variable course while running in the transverse foramens of the cervical vertebrae.

**Case presentation:**

The course of the vertebral artery in the dissected cadaver of a 79 year old female is presented. Dissection of the left vertebral artery showed that the 5^th^ nerve root passes in front of the vertebral artery in the 4^th^ intertransverse space. Further exploration showed that although vertebral artery at first passed at the back of the nerve root it curved downwards again and after passing underneath the 5^th^ nerve root entered the 4^th^ vertebral body. After making a loop in the left half of the vertebrae, vertebral artery ran anterior to the nerve root and after entering the 4^th^ transverse foramen showed up in the 3^rd^ intertransverse space. The shortest distance of the vertebral artery to the midline at the 4^th^ vertebrae level was 4.78 mm.

**Conclusions:**

To our knowledge this case is the first report of a nerve root lying anterior to the vertebral artery in the intertransverse space of the cervical spine. Additionally vertebral artery has never been reported to be so close to the midline. This report signifies the importance of obtaining MRI or contrast enhanced CT scan prior to any cervical spine surgery in the vicinity of the vertebral artery including corpectomies and also careful approach to the intertransverse space during the operation.

## Background

The importance of vertebral artery is because it provides the posterior circulation of the brain and the inner ear. Intraoperative complications of anterior cervical spine surgery include injury to the nerve root, vertebral artery and spinal cord [1]. Vertebral artery laceration has been reported during anterolateral or lateral approach as well as extended lateral decompression of the cervical spine [2]. The incidence of vertebral artery injury during anterior cervical spine procedures is reported to be 0.22–2.77 % [3]. Once injury occurs to the artery hemorrhage is difficult to control and posterior circulation of the brain will be compromised [2]. Such vertebral artery injuries may have severe neurological consequences.

Many landmarks are described to avoid iatrogenic laceration of the vertebral artery such as Longus colli muscle and uncinate process. Vertebral artery shows a considerable degree of variation in its origin, branches, and level of entry into the transverse foramen as well as course and turtosity. This article describes a rare anatomy of vertebral artery–the position of the artery was posterior to the nerve root in the–transverse space. According to our knowledge such case has never been described in the literature before. Considering such variations can limit the incidence of inadvertent vertebral artery injury during surgeries such as corpectomy and foraminotomy. Since this is a cadaveric case report no IRB approval was needed.

## Case presentation

Sixty-four formalin fixed cadavers were dissected in the anatomy lab from November 2007 to January 2008 with an average age of 77 years. The cadavers were decapitated at the level of C1–C2 but the rest of the cervical spine and its muscular attachments were intact. One of these cases presented us with an unusual anatomy of the vertebral artery.

**Fig. 1:**
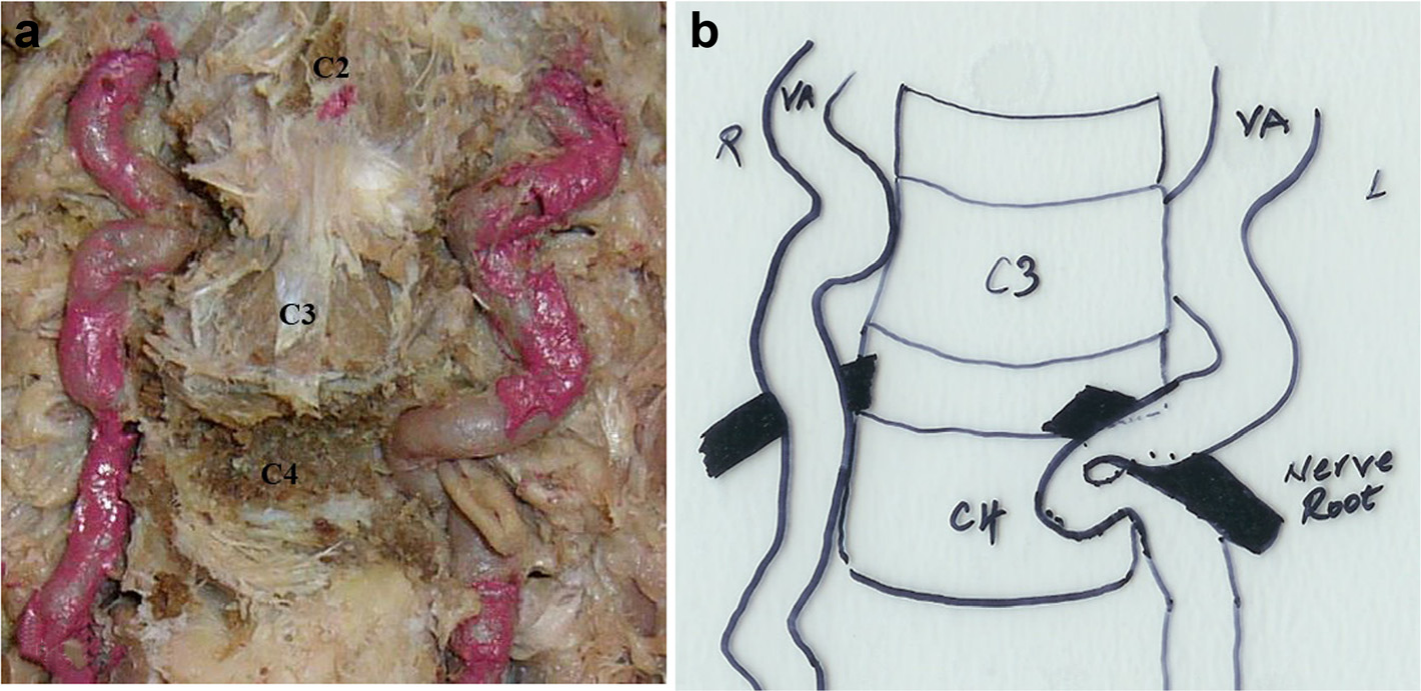
Digital image of the dissected cervical spine and vertebral artery. Complete dissection of the vertebral artery at the *C4* level showed that the artery curves downwards after passing at the back of the nerve root (**a**). Line diagram of the course of the artery on both sides (**b**). *VA* Vertebral artery.

**Fig. 2:**
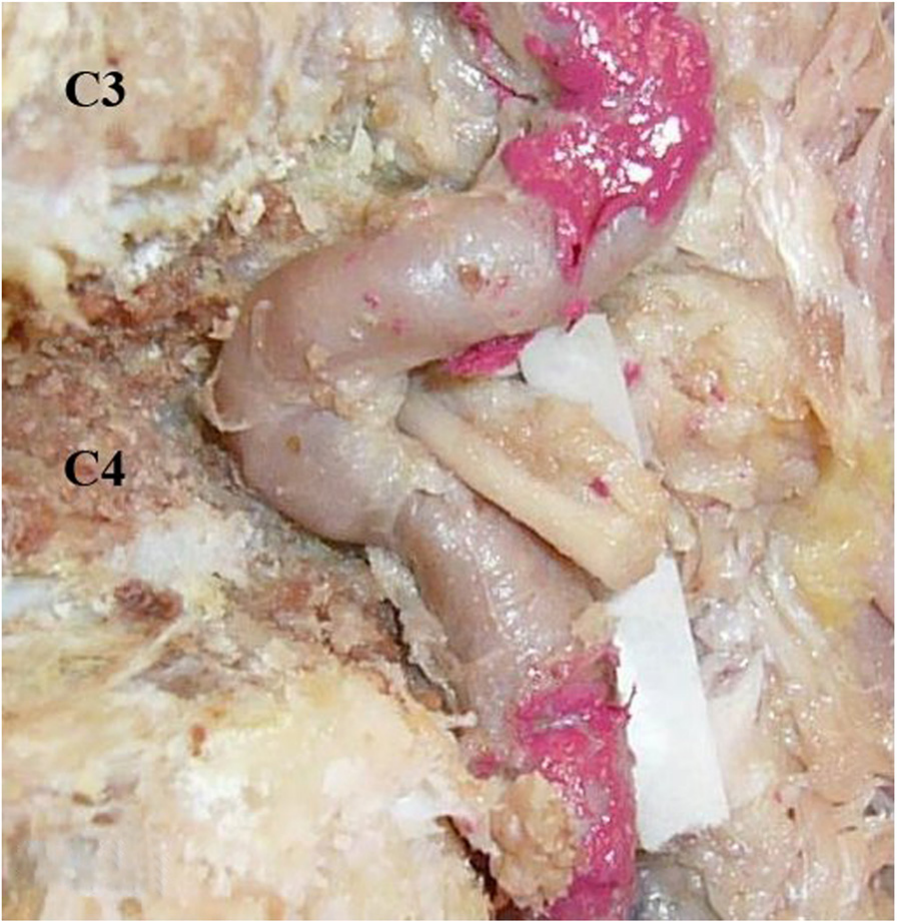
Digital image of the dissected cervical spine and vertebral artery. Complete dissection of the vertebral artery showing its inward loop inside the 4^th^ vertebrae.

The following vertebral artery course was observed in the cervical spine of the cadaver of a 79 year old female. The prevertebral fascia, longus colli muscle and the soft tissue in front of the transverse process and intertransverse space were removed. After removing the intertransverse ligament we found that the most superficial structure in the left 4^th^ intertransverse space was the nerve root. Then the vertebral artery was exposed in all other intertransverse spaces while still running in the transverse foramens. After removing the transverse processes, we found that the artery runs at the back of the 5^th^ left nerve root (Fig. 1). Careful removal of the left side of the 4^th^ vertebral body showed that although vertebral artery at first passed at the back of the nerve root it curved downwards again and after passing underneath the 5^th^ nerve root entered the 4^th^ vertebral body (Fig. 2). After making a loop in the left half of the vertebral, vertebral artery ran anterior to the nerve root and after entering the 4^th^ transverse foramen showed up in the 3^rd^ intertransverse space. The shortest distance of the vertebral artery to the midline in the 4^th^ vertebrae was 4.78 mm.

## Discussion

The uncinate process or the lateral border of the vertebral body have been considered as anatomical landmarks for vertebral artery, however none of them have shown a fixed pattern in the previous studies and the osteophyte formation may render these structures difficult to identify [4]. Moreover the uncinate process can be very close to the vertebral artery due to the cervical spondylosis [1, 5]. Longus colli muscle has also been used as a landmark for vertebral artery, however comparison of the results of different studies show that there is considerable variability in the location of longus colli muscle to the midline [1, 3, 4, 6]. Additionally most of these studies have limited sample size and the results can not be extrapolated.

According to a Pait et al. study, 7.0 % of specimens showed an abnormal level of entrance of the vertebral artery into the transverse foramen, with a level of entrance at C3, C4, C5, and C7 transverse foramen in 0.2 %, 1.0 %, 5.0 %, and 0.8 % respectively. Thirty-one out of 250 patients (12.4 %) had a unilateral anomaly and two (0.8 %) had a bilateral anomaly. They concluded that the turtosity of the vertebral artery can lead to a potentially dangerous medial or a lateral artery displacement. Vertebral artery can loop medially either into a medially enlarged transverse foramen or into the proximal part of the intervertebral foramen [1].

The anterior root of the transverse process is normally 1–2 mm thick. It can be less than 1 mm thick (24 %) or paper thin (16 %), or defective (5 %). These changes make the artery vulnerable to injury from the flakes of transverse process if fractured during the operation [4]. A variation of size can be found in 40 % of people with one artery bigger than the other one.

All the aforementioned variations can impose a considerable risk of injury to the vertebral artery. For example when the artery enters the transverse foramen at a level higher than C6, vertebral artery can be lacerated while removing the longus colli muscle. Our case signifies the importance of obtaining an appropriate imaging study to delineate the course of vertebral artery preoperatively. Magnetic resonance imaging (MRI), contrast enhanced computed tomography (CT) and angiography can all provide such information. The surgeon should suspect penetration of vertebral artery into the vertebrae if there was a defect in the cervical vertebral body on the preoperative CT scan.

Since damage of the nerve root can be as disastrous as laceration of the vertebral artery, the surgeon should be very careful while approaching the intertransverse space. Such penetrations of the vertebral artery into the vertebrae can also complicate the corpectomy in which the artery can be lacerated by high speed burr which mandates review of appropriate imaging studies prior to cervical corpectomies. Since the course of the vertebral artery can be highly variable in the intertransverse space we speculate that the best approach to expose the artery is removal of the transverse process. After that the surgeon can follow the course of the artery. Such variation should also be considered in foraminotomies where the surgeon does not expect the vertebral artery to be present behind the nerve root while doing a posterior foraminotomy. Although the safe limit of corpectomy to the midline is 8 mm on each side it can have disastrous consequences in such inward looping of the vertebral artery.

This case report describes a rare course of vertebral artery located at the back of the nerve root in the intertransverse space and penetration of the vertebral artery into the vertebrae as close as 4.8 mm to the midline. Though this is an unusual entity, surgeons have to be wary of such an anatomy. Further, radiological studies to visualize the course of vertebral artery prior to corpectomies and foraminotomies can be useful in preventing inadvertent arterial injury.

## Conclusion

Vertebral artery can be very close to the midline in subaxial cervical spine. This report signifies the importance of obtaining MRI or contrast enhanced CT scan prior to any cervical spine surgery in the vicinity of the vertebral artery including corpectomies and also careful approach to the intertransverse space during the operation.
